# Editorial: Non-classic congenital adrenal hyperplasia caused with common and rare forms: Unresolved issues and implications on clinical management

**DOI:** 10.3389/fendo.2023.1145352

**Published:** 2023-02-01

**Authors:** Yu Li, Qinjie Tian, Yanping Kuang, Dongzi Yang

**Affiliations:** ^1^ Center for Reproductive Medicine, Sun Yat-sen Memorial Hospital, Guangzhou, China; ^2^ Division of Gynaecologic Endocrinology, Peking Union Medical College Hospital, Beijing, China; ^3^ Division of Reproductive Endocrinology, the 9th affiliated hospital, Shanghai Jiaotong University, Shanghai, China; ^4^ Division of Reproductive Endocrinology, Sun Yat-sen Memorial hospital, Sun Yat-sen University, Guangzhou, China

**Keywords:** congenital adrenal hyperplasia, non-classical type, diagnosis, reproduction, clinical management

The non-classic type of Congenital Adrenal Hyperplasia (NCCAH) is easily to be mis- or undiagnosed. It is characterized mainly by primary amenorrhea/oligomenorrhea/repeated functional ovary cysts/anovulatory cycles/infertility and/or sex hormonal disorders. Multidisciplinary team (MDT) consulting by pediatric, gynecological, or reproductive endocrinologists should be provided to make an etiological diagnosis of various enzyme deficiencies and make a plan of reasonable management.

This Research Topic has focused on diagnosis and differential diagnosis, the characteristics of phenotypes, new genotypes, clinical management, reproduction and assistant reproductive technique (ART) for NCCAH patients. It should be helpful to broaden and widen the understanding about the management of NCCAH patients.

For example, we learn in the review of Adriaansen et al. that the main problem in NCCAH patients is not the inadequate cortisol production, but the increased production of adrenal androgens. Hyperandrogenism can lead to rapid postnatal growth, advanced bone age, and premature pubarche in childhood, as well as acne, hirsutism, menstrual irregularities (in females), and insulin resistance in adulthood. Therefore, NCCAH patients should be carefully evaluated and the advantages and disadvantages of different treatment options used in patients with NCCAH were discussed in this review. In this retrospective observational study of Simon et al., the interaction between muscle-to-fat ratio (MFR) and components of metabolic syndrome in pediatric subjects with NCCAH were explored. It was founded that children and adolescents with NCCAH have a body composition characterized by an imbalance of MFR, while glucocorticoid therapy does not appear to adversely affect their body composition. Lu et al, reported a rare male case of non-classic Lipoid congenital adrenal hyperplasia (LCAH) caused by mutations in the steroidogenic acute regulatory protein (StAR) and reviewed the literature. It showed that the clinical phenotypes of non-classic LCAH are highly variable. Routine physical examination, laboratory measurement, genetic testing, and, importantly, enzymatic activity assay may facilitate the early diagnosis of non-classic LCAH. Yin et al. reported a rare 17- OHD female patient with normal 46,XX karyotype accompanied by ovarian gonadoblastoma with dysgerminoma. Although the tumorigenesis is common in 46 XY 17OHD patient, but it cannot be ruled out in very rare 46 XX CAH patient. Infertility is always one of the main clinical manifestations of partial female 17-OHD patients. Jiang et al. reported two female infertile patients with partial 17-OHD delivered successfully after assisted reproductive treatment and review the literature. It suggested that the pregnancy potentials of infertile partial 17-OHD women seemed to increase with the adoption of IVF-ET. Considering the sustained elevated P4 level, PPOS is a feasible protocol for them in COH. In the systematic review of Guo et al., a meta-analysis was performed to evaluate the pregnancy complications in CAH women caused by rare enzymatic deficiency excluding 21OHD. The result showed that fertility is possible for these patients but special care was necessary when planning, seeking and during pregnancy.

## Author contributions

YL and DY are responsible for initiating and summarizing this Research Topic. QT, YK are guested editor for this Research Topic. All authors contributed to the article and approved the submitted version.

